# Minimal Physiologically-Based Pharmacokinetic (mPBPK) Metamodeling of Target Engagement in Skin Informs Anti-IL17A Drug Development in Psoriasis

**DOI:** 10.3389/fphar.2022.862291

**Published:** 2022-04-25

**Authors:** Vivaswath S. Ayyar, Jong Bong Lee, Weirong Wang, Meghan Pryor, Yanli Zhuang, Thomas Wilde, An Vermeulen

**Affiliations:** ^1^ Janssen Research & Development, LLC, Spring House, PA, United States; ^2^ Janssen R & D, Division of Janssen Pharmaceutica NV, Beerse, Belgium

**Keywords:** IL-17A, MBMA, mPBPK, psoriasis, target engagement, translational medicine, secukinumab, ixekizumab

## Abstract

The pharmacologic effect(s) of biotherapeutics directed against soluble targets are driven by the magnitude and duration of free target suppression at the tissue site(s) of action. Interleukin (IL)-17A is an inflammatory cytokine that plays a key role in the pathogenesis of psoriasis. In this work, clinical trial data from two monoclonal antibodies (mAbs) targeting IL-17A for treatment of psoriasis (secukinumab and ixekizumab) were analyzed simultaneously to quantitatively predict their target engagement (TE) profiles in psoriatic skin. First, a model-based meta-analysis (MBMA) for clinical responses was conducted separately for each drug based on dose. Next, a minimal physiologically-based pharmacokinetic (mPBPK) model was built to assess skin site IL-17A target engagement for ixekizumab and secukinumab simultaneously. The mPBPK model captured the observed drug PK, serum total IL-17A, and skin drug concentration-time profiles reasonably well across the different dosage regimens investigated. The developed mPBPK model was then used to predict the average TE (i.e., free IL-17A suppression) in skin achieved over a 12-weeks treatment period for each drug following their respective regimens and subsequently assess the TE-efficacy response relationship. It was predicted that secukinumab achieved 98.6% average TE in the skin at 300 mg q4w SC while ixekizumab achieved 99.9% average TE under 160 mg (loading) followed by 80 mg q2w SC. While direct quantification of free IL-17A levels at the site of action is technically challenging, integrated mPBPK-MBMA approaches offer quantitative predictions of free IL-17A levels at the site of action to facilitate future drug development via IL-17A suppression in psoriasis.

## Introduction

Psoriasis is a chronic autoimmune and inflammatory skin disease characterized by red, itchy, and scaly skin patches. According to the World Psoriasis Day consortium, psoriasis affects over 125 million people worldwide (https://www.psoriasis.org/psoriasis-statistics/; date accessed: 03/05/2022). Many cytokines and immune cells have been identified to promote the disease initiation and propagation (Schon and Boehncke, 2005); among those, the Interleukin (IL)-23/T helper (Th)17/IL-17 immune pathways play pivotal roles (Lima and Kimball, 2010). IL-17A is an inflammatory cytokine secreted by Th17 cells and is reported to play a key role in the pathogenesis of psoriasis (Krueger et al., 2012; Martin et al., 2013). In psoriatic patients, both IL-23 and IL-17 exhibit elevated expression in lesional skin sites (Arican et al., 2005). Upon release, IL-17A signals various cells in the skin, such as keratinocytes, which are activated to produce downstream mediators reported to be elevated in psoriatic skin (Harper et al., 2009; Brembilla et al., 2018). Drugs targeting the IL-17A cytokine and its signaling pathway have shown effectiveness in treatment of psoriasis and other immune disorders (Martin et al., 2013; Brembilla et al., 2018). There are two marketed monoclonal antibodies (mAbs) that specifically target the IL-17A cytokine: secukinumab and ixekizumab. Both compounds offer safe and effective treatment for psoriasis patients (Blauvelt, 2016; Frieder et al., 2018).

The pharmacologic effect(s) of biotherapeutics directed against soluble targets are driven by the magnitude and duration of free target suppression at the site of action *in vivo* (Zheng et al., 2015; Chen et al., 2016; Ayyar et al., 2021a). However, the relationship between *in vivo* target/pathway blockade at the target site and clinical improvement in disease severity warrants further study. Population PK models of diverse mAbs, including anti-IL17A mAbs (secukinumab and ixekizumab), using serum total PK data in psoriasis patients have been reported. Further, exposure-response and/or semi-mechanistic PK/PD models linking mAb PK to PD endpoints have been established (Zhu et al., 2009; Bruin et al., 2017; Yao et al., 2018; Jackson et al., 2022). However, relationships developed using such models may not be readily extrapolated to other therapies against the same target, owing to differing PK/biodistribution characteristics, target binding affinities, and/or biophysical properties. Knowledge of the magnitude and duration of target engagement required to achieve the desired therapeutic benefit can be useful to facilitate discovery and development of future therapies. The minimal physiologically-based pharmacokinetic (mPBPK) model, first proposed by Cao and Jusko (Cao et al., 2013), is a commonly used approach to quantitatively assess the drug exposure and target engagement at the tissue site of action. A typical mPBPK model for a mAb comprises a central (plasma) compartment, lumped “tight” and “leaky” compartments (assigned based on tissue vascular endothelial permeabilities), and a lymph compartment connected by lymphatic flow. It inherits the key advantages of a whole-body PBPK model by using physiologically relevant parameters while focusing only on the tissue of interest hence being easier to implement (Ayyar and Jusko, 2020). With specific tissue PK and target dynamics data to inform the model, target-mediated drug disposition (TMDD) kinetics can be incorporated into the central circulation and/or the specific tissue compartments, as exemplified previously in preclinical studies (Chen et al., 2016; Chen et al., 2018; Zheng et al., 2020a). To our knowledge, an approach integrating mPBPK modeling of human PK and IL-17A TE data with observed clinical response (e.g., disease score) has not been reported thus far.

The present work sought to develop a mPBPK model to predict free IL-17A neutralization in skin for secukinumab and ixekizumab and quantitatively relate it with clinical response rates based on 75% reduction in the Psoriasis Area and Severity Index (PASI) score (PASI75) and 90% reduction in PASI score (PASI90). To this end, clinical trial data from both antibodies were analyzed using model-based meta-analysis (MBMA) coupled with drug-target binding affinity, clinical PK, IL-17A TE with relevant physiological parameters (using mPBPK-TE modeling) to quantitatively predict TE needed to inform future drug development targeting the IL-17A pathway.

## Materials and Methods

### Data Source

All data used in this study were collected from published literature or data published within regulatory reviews. Data from placebo-controlled randomized clinical trials conducted in psoriasis patients evaluating clinical response were included (Table 1). In addition, a phase 1 exploratory study measuring skin biodistribution was also included (Leonardi et al., 2012; Papp et al., 2013; Rich et al., 2013; Langley et al., 2014; FDA, 2015; Mrowietz et al., 2015; Dragatin et al., 2016; FDA, 2016; Papp et al., 2018).

**TABLE 1:** Randomized placebo-controlled clinical trials of secukinumab and ixekizumab in psoriasis patients included in the analyses.

Study Description	Treatment Groups (up to week 12)	N*	References
*Secukinumab*			
Phase II			
Proof of Concept	3 mg/kg IV single dose	36	FDA (2015)
	Placebo		
Low dose-ranging	25 mg SC q4w	125	Papp et al., 2013
	75 mg SC q4w		
	150 mg SC q4w		
	Placebo		
High dose-ranging	3 mg/kg IV single dose	100	Rich et al., 2013
	10 mg/kg IV single dose		
	10 mg/kg IV Wk 0, 2, 4		
	Placebo		
Dose regimen finding	150 mg SC single dose	404	Rich et al., 2013
	150 mg SC q4w		
	150 mg SC Wk 0, 1, 2, 4		
	Placebo		
Phase III			
ERASURE	150 mg SC Wk 0, 1, 2, 3, 4; q4w	734	Langley et al., 2014
FIXTURE	300 mg SC Wk 0, 1, 2, 3, 4; q4w	974	Langley et al., 2014
FEATURE	Placebo	176	FDA (2015)
JUCTURE		181	FDA (2015)
*Ixekizumab*			
Phase I			
Dose-escalation	15 mg IV at Wk 0, 2, 4	46	FDA (2016)
	5 mg SC at Wk 0, 2, 4		
	15 mg SC at Wk 0, 2, 4		
	50 mg SC at Wk 0, 2, 4		
	150 mg SC at Wk 0, 2, 4		
	Placebo		
Phase II			
Dose-ranging	10 mg SC at Wk 0, 2, 4, 8	141	Leonardi et al., 2012
	25 mg SC at Wk 0, 2, 4, 8		
	75 mg SC at Wk 0, 2, 4, 8		
	150 mg SC at Wk 0, 2, 4, 8		
	Placebo		
Phase III			
UNCOVER-1	160 mg SC at Wk 0; 80 mg SC q2w	1,296	Papp et al., 2018
UNCOVER-2	160 mg SC at Wk 0; 80 mg SC q4w	866	
UNCOVER-3	PBO	964	

*Excludes participants enrolled in active comparator arms.

### Model-Based Meta-Analysis

MBMA was performed by non-linear regression of the trial level data. A 75% or greater reduction in the PASI score (PASI75) and 90% or greater reduction in PASI score (PASI90) after 12 weeks of treatment were used as clinical efficacy measurements as these were the common endpoints for secukinumab and ixekizumab. The response rate was adjusted by subtracting the placebo response from the active drug arm response for each study. A dose-based MBMA was conducted using the total dose administered during the 12 weeks divided by the total duration (12 weeks) to obtain dose per unit time (mg/week). Additionally, a TE-based MBMA was conducted based on the average inhibition of IL-17A during the 12 weeks based on the simulation.

The equation used for the MBMA model was:

$$Response=E_{0}+\frac{x^{hill}\cdot \left(E_{max}-E_{0}\right)}{x^{hill}+E_{50}^{hill}}$$ (1)

where, *x* is dose or TE, *E*_*0*_ is baseline response, *E*_*max*_ is maximum response, and *E*_*50*_ is dose or TE needed for 50% of maximum response and *hill* is the Hill coefficient. This MBMA fitted a trendline that described the overall trend of the clinical datasets (see Figures 2, 6).

### Minimal Physiologically-Based Pharmacokinetic–Target Engagement Model

**FIGURE 1:**
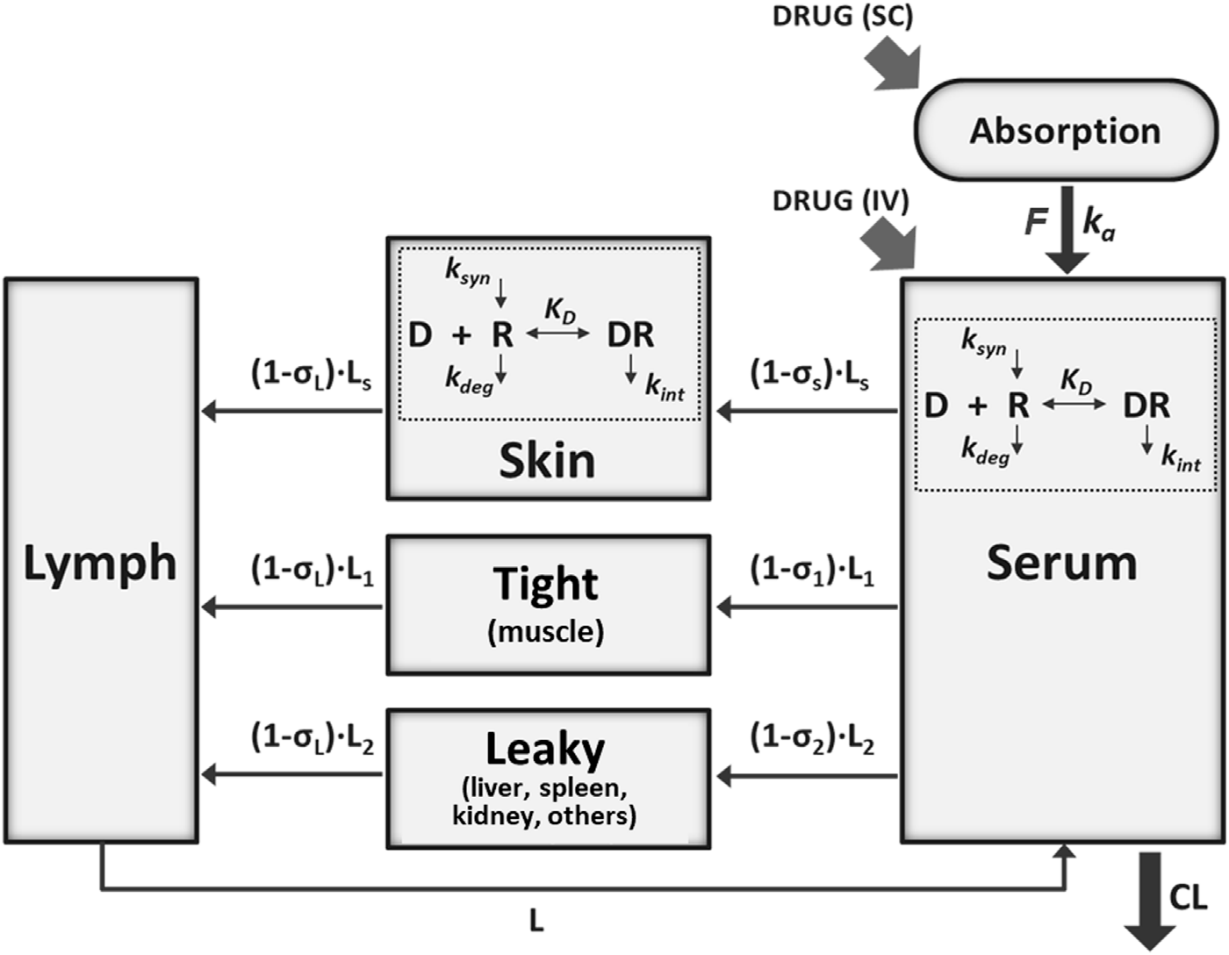
Schematic of the minimal physiologically-based pharmacokinetic (mPBPK) model for secukinumab and ixekizumab incorporating IL-17 binding in serum and skin. See Table 2 for model parameter values.

A mPBPK model incorporating features of TMDD (Chen et al., 2018) was adapted to characterize the interrelationships between secukinumab/ixekizumab and free and total IL-17 in psoriasis patients. The model sought to describe the whole-body physiology and drug-specific characteristics in a minimal manner (Figure 1). Compartments representing serum, lymph, skin, muscle, and an absorption depot were incorporated. Other organs were lumped into a “leaky” compartment, where “leaky” refers to the permeability of the vascular endothelial structure of these tissues. Since skin and muscle together account for ∼90% of total body mass of “tight” organs, an additional “tight” compartment lumping other organs was excluded. Circulation of secukinumab and ixekizumab was assumed to depend predominantly on lymphatic flow and paravascular convection (Cao et al., 2013). Although interaction between the antibody and IL-17A would occur ubiquitously, this process was simplified to occur in skin and serum compartments only (Zheng et al., 2020a). Similarly, distributary circulation of IL-17A and the drug-target complex were not modeled explicitly; instead, their dynamics in serum and in skin were considered locally (Chen et al., 2018). The elimination rate constant for free ligands, especially soluble cytokines, is typically substantially faster than that of mAb-bound ligand (Davda and Hansen, 2010). As such, when ligand becomes bound to the mAb and takes on the disposition properties of the mAb, there is a rapid and measurable increase in total ligand concentrations (Davda and Hansen, 2010). Consistently, identical elimination rate constants for mAb-IL17A complexes and free mAb were assumed to jointly characterize serum total mAb and total target (free IL-17A+ complex) concentrations. Equations describing the model are listed in Supplementary Materials. A naïve pooled approach was used for model fitting, as available data from trial reports were at a group level. For model simulation, the population mean of the parameter estimates was used. Clinically approved dose regimens of secukinumab and ixekizumab were simulated and free IL-17A in skin was predicted as a measure of target engagement for each drug.

### Data Analysis and Software

The data were extracted as mean values from published graphs by computer digitization (WebPlotDigitizer, version 4.1; https://automeris.io/WebPlotDigitizer). NONMEM version 7.4 (Icon Development Solutions, Ellicott City, MD, United States) was used for mPBPK model fitting and parameter estimation. The model code is provided as Supplementary Material. The First-Order Conditional Estimation method with Interaction (FOCE-i) was applied. Since mean data were analyzed, inter-individual variability (IIV) was not considered and the variance–covariance matrix was fixed to 0. A proportional error model was used to describe the residual error. Model performance was evaluated by goodness-of-fit plots and objective function values (OFV). MBMA and mPBPK model simulations were performed in R (Comprehensive R Network version 3.6.3 [www.r-project.org]). The non-linear least squares function (nls) provided in the stats package (version 3.6.3) was used for MBMA and the mrgsolve package (version 0.10.0) was used for mPBPK model simulations.

## Results

### Model-Based Meta-Analysis Based on Secukinumab and Ixekizumab Doses

**FIGURE 2:**
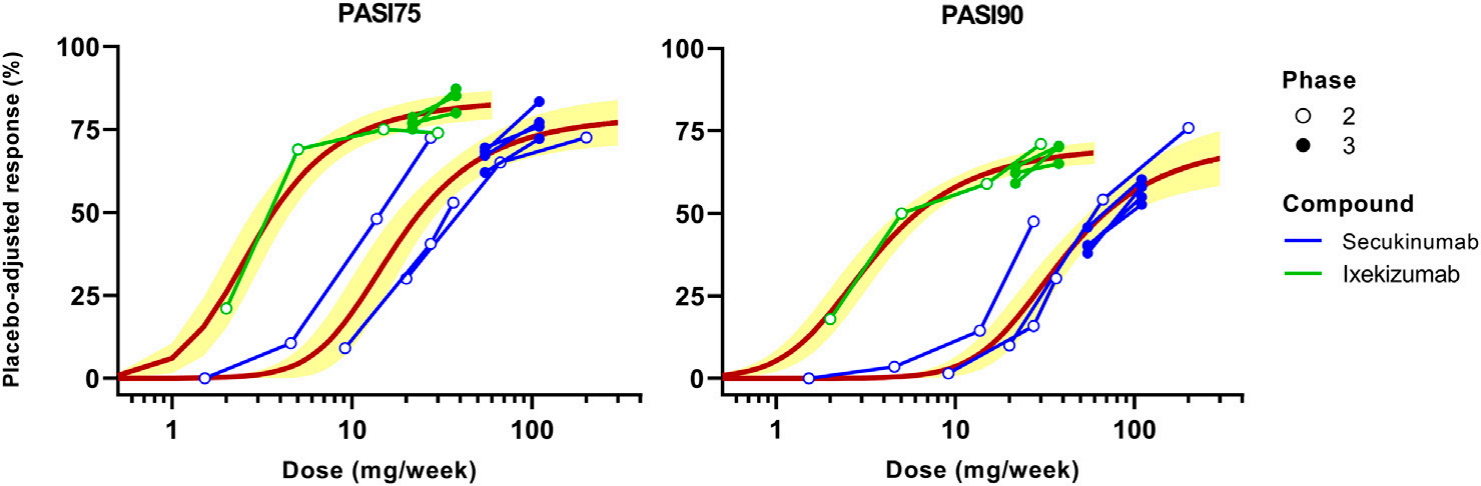
Dose-based model-based meta-analysis (MBMA) of clinical trial results for secukinumab and ixekizumab. Average dose (mg/week) was calculated based on total administered dose over 12 weeks. Clinical response 12 weeks after the initial dose was used as efficacy endpoint. Solid lines represent model-fitted trendline, whereas symbols indicate observed mean data. The observed mean data from individual clinical studies are connected. Shaded bands depict the 95% CI around the regression curves.

MBMA conducted for secukinumab and ixekizumab based on dose (mg/week) is shown in Figure 2. Two dose regimens of secukinumab are approved for the treatment of psoriasis: 150 or 300 mg SC at Weeks 0, 1, 2, 3 followed by q4w from Week 4 onwards. The MBMA showed that the 300 mg dose regimen yielded higher PASI75 and PASI90 responses than the 150 mg dose regimen. Moreover, the trendline constructed by model fitting predicted that there may be a continuously increasing trend when the dose is increased even further from the 300 mg dose regimen, which is more apparent with the more stringent endpoint of PASI90.

For ixekizumab, only one dose regimen is approved for the treatment of psoriasis: 160 mg SC at Week 0 followed by 80 mg SC q2w from Week 2 onwards. Four different dose levels (10, 25, 75 or 150 mg SC at Week 0, 2, 4 and 8) were tested in the Phase 2 study, which showed obvious dose-response for both PASI75 and PASI90 (Figure 2). Phase 3 studies of ixekizumab tested both 80 mg SC q4w and q2w following the initial induction dosing for which the results are shown. The q2w regimen yielded slightly higher PASI75 and PASI90 responses. Nonetheless, the fitted trendline indicated that further increasing the dose may achieve marginally higher efficacy. Note that the dose-response curves for secukinumab and ixekizumab do not overlap, signifying the involvement of additional determinants (beyond mAb dose) that may be incorporated to jointly model the dose-exposure-TE-clinical response relationship for these therapies.

### Minimal Physiologically-Based Pharmacokinetic Modeling to Assess IL-17A TE

**FIGURE 3:**
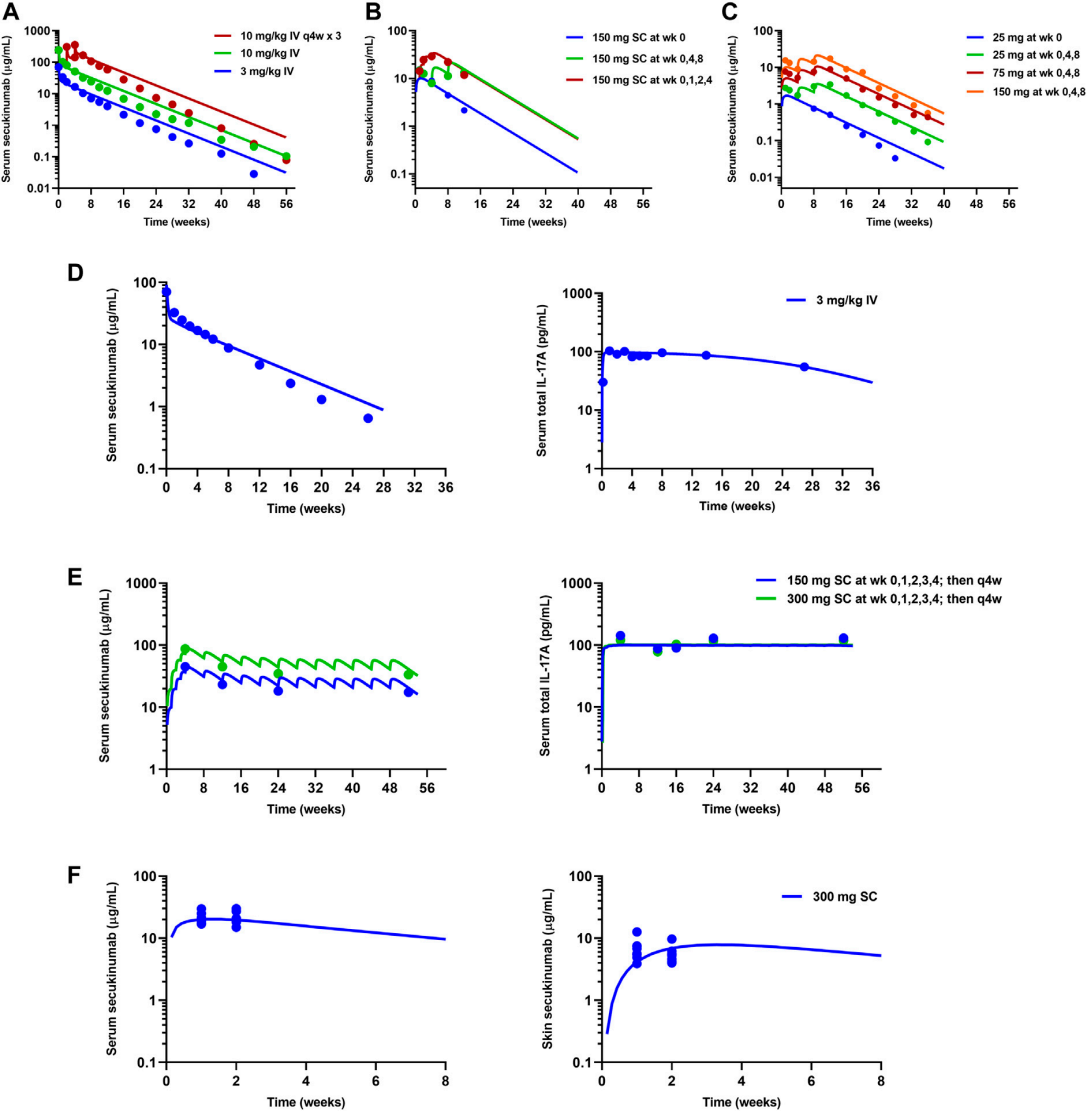
Model fitting of observed mean data in clinical trials for secukinumab. Refer to Table 1 for details on each clinical study. **(A)** High dose-ranging study (Rich et al., 2013). **(B)** SC dose regimen-finding study (Rich et al., 2013). **(C)** SC low dose-ranging study (Papp et al., 2013). **(D)** Proof-of-concept study (FDA, 2015). **(E)** Phase 3 studies (Langley et al., 2014; FDA, 2015). **(F)** Exploratory biodistribution study (Dragatin et al., 2016). Solid lines represent model-fitted profiles, whereas symbols indicate observed mean data.

The mPBPK model for secukinumab and ixekizumab IL-17A TE was developed by fitting data reported from clinical trials conducted in psoriasis patients (Table 1). The model simultaneously captured the observed secukinumab serum concentration-time profiles following IV (3 and 10 mg/kg) and SC (25–300 mg) doses across various dosing regimens (Figures 3A–C). In the secukinumab proof-of-concept study (FDA, 2015), both serum secukinumab PK and serum total IL-17A (i.e., antibody-bound IL-17A and free IL-17A) were measured and well-characterized by the model (Figures 3D,E). In an exploratory biodistribution study (Dragatin et al., 2016), both serum and skin secukinumab concentrations were profiled. The mPBPK model simultaneously captured these observed data well (Figures 3F,G).

**FIGURE 4:**
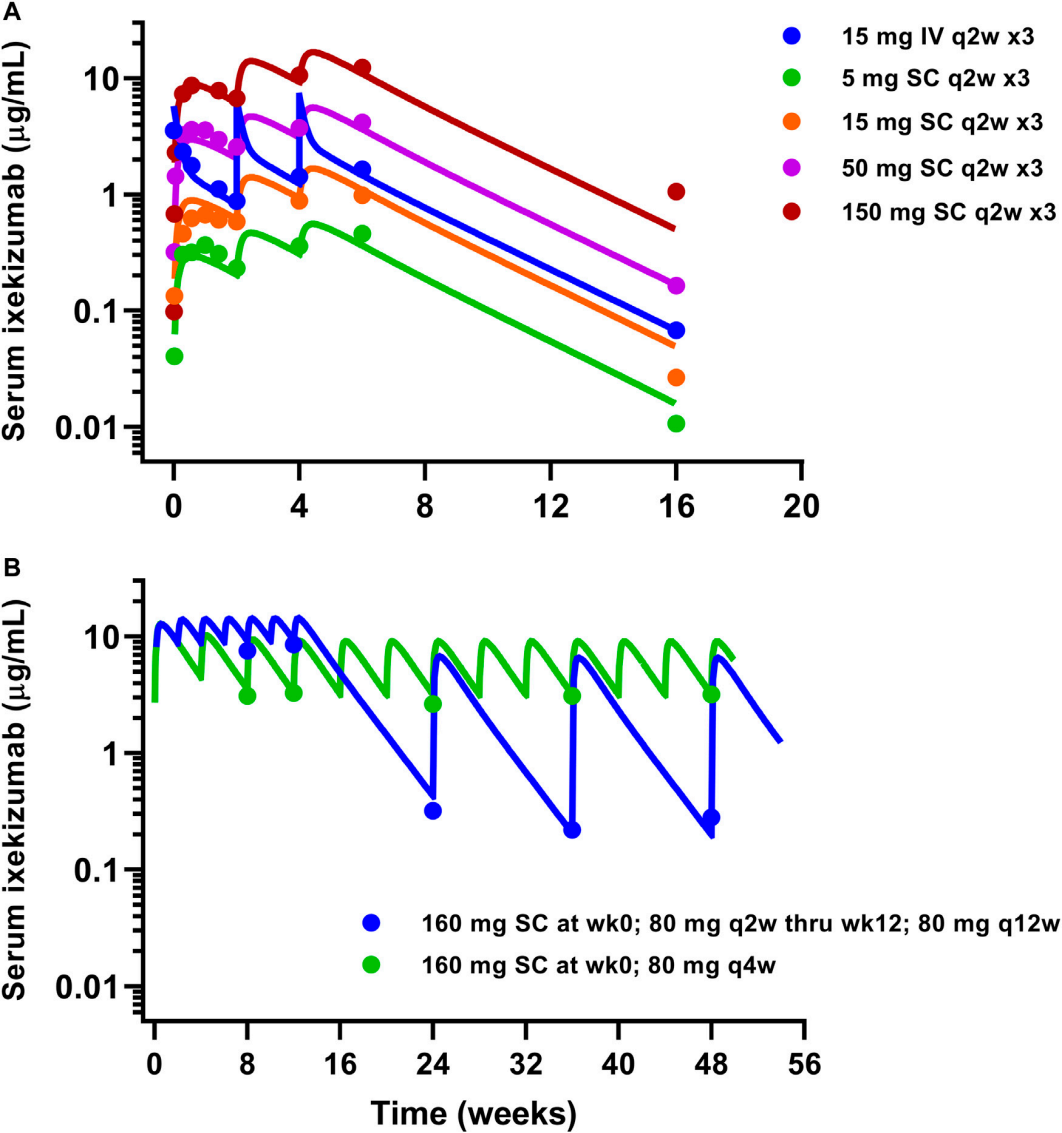
Model fitting of observed ixekizumab mean serum PK data. **(A)** Phase 1 study (FDA, 2016). **(B)** Phase 3 studies (Papp et al., 2018). Solid lines represent model-fitted profiles, whereas symbols indicate observed mean data.

Similarly, available serum ixekizumab concentration-time profiles were also well captured by the mPBPK model (Figure 4). The physiological flows/volumes, parameters relating to IL-17A dynamics, and drug-target binding affinities were fixed to reported values as summarized in Table 2. Parameter estimates for SC bioavailability (F) and the first-order absorption rate constant (k_a_) for secukinumab and ixekizumab were fixed based on population PK model-derived estimates (Bruin et al., 2017; Jackson et al., 2022), whereas free mAb clearances (CL) were re-estimated using the mPBPK model and were found to be reasonably similar to previous estimates of total CL for secukinumab (0.19 L/day) and ixekizumab (0.29 L/day) (Bruin et al., 2017; Jackson et al., 2022). Parameters related to IL-17A were kept identical between secukinumab and ixekizumab. The elimination rate constants for free IL-17A in serum and skin were fixed to values determined previously (Zheng et al., 2020b). Consistent with physiological expectation, the elimination rate constant for free IL-17A in serum was much faster compared to that in skin (Table 2). Selected parameters such as free mAb CL, elimination rate constant for serum total IL-17A (i.e., secukinumab-bound IL-17A and free IL-17A) (k_int_), and reflection coefficients for skin (σ_s,skin_) and leaky compartments (σ_2,leaky_) were estimated upon simultaneous fitting of all observed data with acceptable precision (Table 2).

**TABLE 2:** Summary of mPBPK model parameters and estimates.

Parameter		Unit	Value (RSE%)^a^		Source^c^
			Secukinumab	Ixekizumab	
Clearance (*CL*)		L/day	0.154 (3.3%)	0.379 (8.0%)	
SC Bioavailability (*F*)		—	0.729	0.81	[1, 9, 10]
Absorption rate constant (*k*_*a*_)		1/day	0.18	0.24	[1, 9, 10]
Lymph flow rate	***L*,** total	L/day	2.9		[2]
	***L***_***s***_ **,** skin	L/day	0.247		[2, 3]
	***L***_***1***_ **,** muscle	L/day	0.71		[2, 3]
	***L***_***2***_ **,** leaky tissue	L/day	1.943		[2]
Volume of distribution	***V***_***p***_ **,** plasma	L	2.6		[2]
	***V***_***2***_ **,** leaky tissue	L	4.37		[2]
	***V***_***s***_ **,** skin	L	1.81		[2, 3]
	***V***_***1***_ **,** muscle	L	6.3		[2, 3]
	***V***_***L***_ **,** lymph	L	2.6		[2]
*k*_*deg*_ IL-17A	**Skin**	1/day	2.44		k_deg,skin_ = k_syn_/baseline_skin_
	**Plasma**	1/day	45.5		[4]
*k*_*int*_ complex	**Skin**	1/day	0.34		2.5 x Ls/Vs. [5]
	**Plasma**	1/day	1.24 (6.4%)	1.24^b^	
baseline IL-17A	**Skin**	pM	0.28		[6]
	**Plasma**	pM	0.015		[6]
*k*_*syn*_ IL-17A		pM/day	0.683		k_syn_ = k_deg,plasma_ x baseline_plasma_
*K*_*D*_		pM	129	1.8	[7]
Reflection co-efficient	***σ***_***s***_ **, skin**	—	0.630 (7.0%)	0.63^b^	
	***σ***_***1***_ **, muscle**	—	0.95		[2, 8]
	***σ***_***2***_ **, leaky**	—	0.363 (14.2%)	0.524 (17.4%)	
	***σ***_***L***_ **, lymph**	—	0.2		[2]

a Parameters estimated by mPBPK, modeling are indicated with RSE% shown in parentheses.

b Parameter assumed to be the same as for secukinumab.

c References: [1] (Bruin et al., 2017); [2] (Cao et al., 2013); [3] (Shah and Betts, 2012); [4] (Zheng et al., 2020); [5] (Chen et al., 2016); [6] (Dragatin et al., 2016); [7] (Adams et al., 2020); [8] (Chen et al., 2018); [9] (FDA, 2015); [10] (FDA, 2016).

**FIGURE 5:**
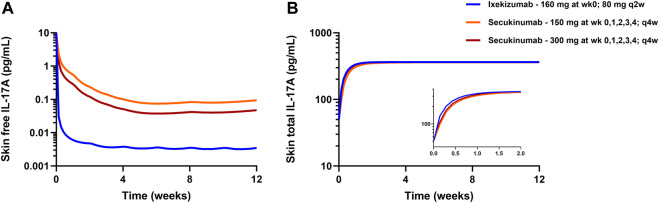
mPBPK model-predicted free IL-17A lowering **(A)** and total IL-17A **(B)** in psoriatic skin following secukinumab or ixekizumab. Solid lines represent model simulations.

The developed mPBPK model was used to simulate free IL-17A suppression in skin following approved dose regimens of secukinumab and ixekizumab. The simulated profiles in Figure 5A show that free IL-17A concentrations were lowered by 2-3 orders of magnitude following treatment with secukinumab or ixekizumab, and greater skin free IL-17A suppression is predicted after ixekizumab treatment, driven by its higher binding affinity for IL-17A compared with secukinumab (Table 2). Consistently, skin concentrations of total (free + bound) IL-17A increased substantially above baseline, reaching a steady-state maximum around 2–3 weeks following either secukinumab or ixekizumab at their respective doses (Figure 5B).

### Target Engagement Model-Based Meta-Analysis

**FIGURE 6:**
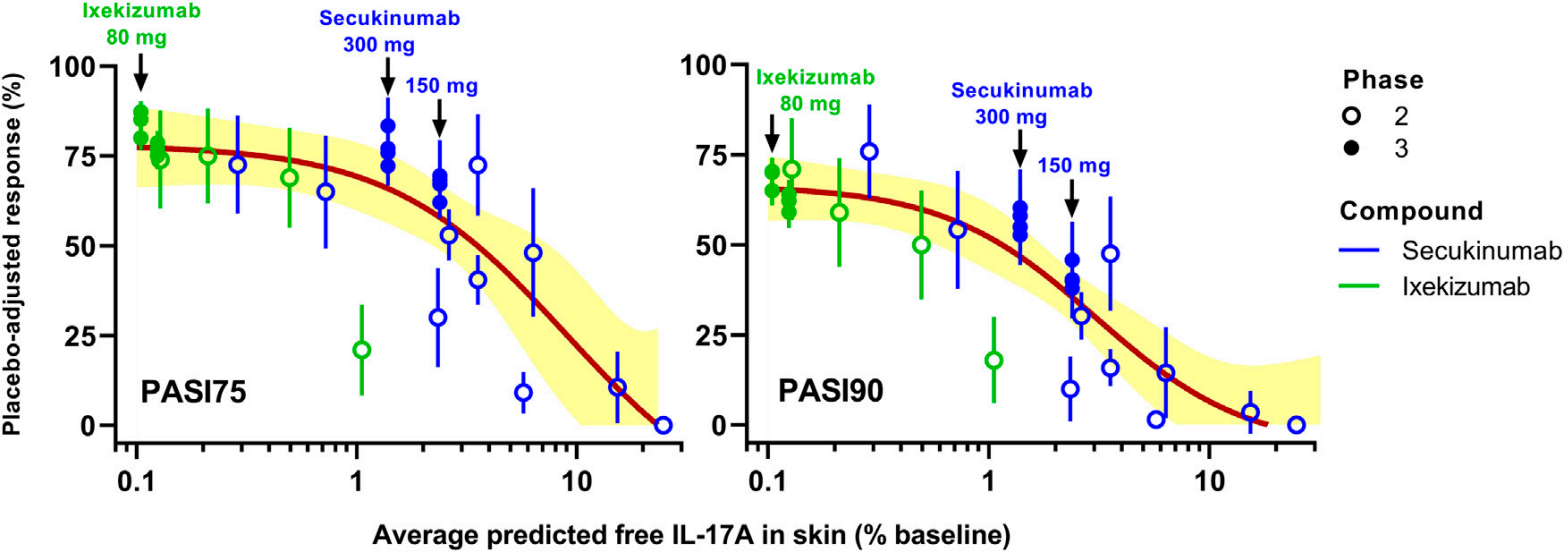
Simulated target engagement (TE)-based model-based meta-analysis (MBMA) of clinical trial results for secukinumab and ixekizumab. Average TE was calculated based on the concentration of free IL-17A over 12 weeks. Clinical response 12 weeks after the initial dose was used as efficacy endpoint. The two approved dose regimens of secukinumab and the approved dose regimen of ixekizumab are marked with arrows on the graphs. Solid lines represent model-fit, whereas symbols indicate observed placebo-adjusted clinical responses. Shaded bands depict the 95% CI around the TE-PASI regression curves.

A TE-based MBMA was subsequently conducted based on the predicted average free IL-17A lowering in skin over 12 weeks, accounting for differences in dose regimen (doses, frequencies, and induction dosing), drug PK, and drug-target binding affinity between secukinumab and ixekizumab. Figure 6 depicts the MBMA results based on PASI75 (left) and PASI90 (right) for both secukinumab and ixekizumab. Of interest, unlike the dose-based MBMA, the same relationship between skin free IL-17A suppression, and clinical efficacy could be applied successfully to the majority of both secukinumab and ixekizumab data. The approved dose regimen of secukinumab (300 mg q4w SC) was predicted to achieve 98.6% average TE over 12 weeks. The approved dose regimen of ixekizumab (160 mg at week 0 followed by 80 mg q2w SC) was predicted to achieve 99.9% average TE over 12 weeks.

## Discussion

This work aimed to quantitatively identify the extent of TE of IL-17A in skin needed to achieve a clinical response comparable to existing antibody therapies (i.e., secukinumab and ixekizumab). MBMA of clinical trial data for two marketed anti-IL-17A drugs was coupled with mPBPK modeling to understand the relationship between IL-17A suppression in skin and clinical efficacy in psoriasis patients. Routinely employed population-based PK and exposure-response models typically relate total serum or plasma concentrations to clinical responses (Bruin et al., 2017; Jackson et al., 2022). Such models are highly useful for informing dose optimization, quantifying inter-subject variability, and identifying clinically meaningful covariates (Ayyar and Jusko, 2020). However, it is well recognized, for neutralizing agents, that additional factors such as tissue biodistribution and target binding affinity are major determinants governing the magnitude and duration of free target suppression (and relatedly, clinical efficacy) at the site of action. Incorporating such aspects using a mPBPK-TE-MBMA modeling approach, a main focus of this effort, is expected to strengthen the predictability of clinical response across different IL-17-targeting agents.

First, MBMA was conducted based on average dose administered per week (mg/week) over 12 weeks (Figure 2). The analysis showed that dose regimens of secukinumab may not have reached the plateau, especially for the more stringent endpoint of PASI90. However, ixekizumab appeared to have approached the plateau of the dose-response curve. The dose-based MBMA results are difficult to interpret across anti-IL-17A drugs due to different characteristics including differing dose regimens, binding affinities, and pharmacokinetics.

To address this challenge, a mPBPK model was developed and applied to account for the different characteristics of the two mAbs and predict the tissue site TE achieved by each drug at their respective dose regimens. Our groups have demonstrated the utility of mPBPK and mechanistic PK-TE-PD models for various biotherapeutics and targets with preclinical and translational applications (Chen et al., 2016; Chen et al., 2018; Jiang et al., 2020; Zheng et al., 2020a; Ayyar et al., 2021a; Ayyar et al., 2021b). In this study, a combined mPBPK-MBMA approach was taken to jointly examine clinical data from Phase 1-3 studies of secukinumab and ixekizumab. The drug- and system-parameters were either fixed based on reported values or were estimated based on observed clinical data including serum and skin drug concentrations and serum total IL-17A concentrations (Table 2).

The skin site IL-17A TE predicted by the mPBPK model was combined with the MBMA for secukinumab and ixekizumab (Figure 6). Clear trends were observed between both PASI75 and PASI90 and IL-17A TE (% lowering of free IL-17A from baseline) for secukinumab and ixekizumab together. The 300 mg SC dose regimen of secukinumab achieved less IL-17A TE compared to ixekizumab. Consistently, it appeared to have reached near-maximal effect for PASI75 but not for PASI90. On the other hand, the approved dose regimen of ixekizumab was predicted to reach 99.9% TE, i.e., lowering the free IL-17A to 0.1% of baseline, and ixekizumab was reported to have reached higher PASI75 and PASI90 responses. The TE predicted by the mPBPK modeling identified the TE differences for the two drugs: 98.6% average TE was predicted for the 300 mg dose regimen of secukinumab and 99.9% average TE for ixekizumab over a 12-weeks treatment period, and this implies that skin site free IL-17A was lowered to 1.4 and 0.1% of the baseline, respectively. Of note, in contrast with Figure 2, where the PASI response was plotted against mAb doses, a single nonlinear sigmoidal function (Eq. 1) related PASI response and free IL-17A in skin to adequately capture the majority of Phase 2 and Phase 3 response data collected for both mAbs. This confirms that the PASI/IL-17A relationship should be mAb-independent, and that the minor deviations are likely due to uncertainties in skin free IL-17 prediction. This finding exemplifies the mechanistic insight gained using the proposed mPBPK-TE approach over the more empirical MBMA based on dose alone, which required separate relationships to analyze secukinumab and ixekizumab data independently.

While the developed mPBPK model with TMDD kinetics provided a good description of the observed data, it is important to note some limitations and assumptions in the current approach. First, there are no tissue site IL-17A TE data being reported and very limited serum TE data to support the mPBPK model development; therefore, model estimation of IL-17A target related parameters (including turnover and route of clearance) may still possess some uncertainty. Hence, relative differences in the model-predicted free IL-17A lowering in skin between secukinumab and ixekizumab may be more reliable for decision-making compared to absolute predicted values. The estimation of drug-specific reflection coefficients (σ_2,leaky_) between secukinumab and ixekizumab was based on observed PK data for both compounds. There could be a mechanistic reason for the difference, e.g., differences in charge or IgG subtype, which could impact capillary permeability or tissue binding resulting in an apparent difference in σ_2, leaky_ between secukinumab and ixekizumab. Though potential impacts from other technical issues, e.g., PK assay or sampling times cannot be ruled out, the correlation between PASI and model predicted free IL-17A (Figure 6) supported the overall credibility of the model estimates. Fixing a majority of mPBPK parameter values *a priori* enables the model to predict tissue disposition but confers less flexibility to best characterize or “fit” multiple serum PK datasets simultaneously. Such constraints imposed on one or more parameters could have contributed to the modest underestimation of serum concentrations at early time points following IV secukinumab. (Figure 3D). The model assumes that the production of IL-17A remains constant throughout the course of treatment. However, since many biomarkers, including Th17 cells (Aguilar-Flores et al., 2020), IL-17 signature genes (Chiricozzi et al., 2016), and serum IL-17 (Chen et al., 2013) are positively correlated with disease severity, this assumption may not hold true, especially upon long-term treatment. In the TE-based MBMA, results from a small number of secukinumab and ixekizumab Phase 2 studies deviated notably from the overall PASI75/90-TE relationship. Though the exact cause of these deviations is not known, potential differences in target dynamics as a function of treatment duration may have contributed. Tissue-specific ISF turnover rates can impact the binding kinetics between antibodies and soluble targets to varying degrees, depending upon their rate constants of association (*k*_*on*_) and dissociation (*k*_*off*_) (Li et al., 2018). This work involved the comparison of the target binding profiles of two approved mAbs (with broadly differing affinities) within skin, a tissue with relatively higher ISF turnover rate (Li et al., 2018). Interestingly, Li et al. showed that both secukinumab and ixekizumab possess substantially fast *k*_*on*_ rates (4.1 × 10^5^ 1/Ms and 7.5 × 10^6^ 1/Ms, respectively) (Liu et al., 2016), which is favored for targeting within high ISF turnover tissues. This may reconcile why the current model, despite assuming equilibrium-based binding, performed satisfactorily in relating PASI response with predicted % free IL-17A for both drugs jointly. Nonetheless, tissue-specific ISF turnover and drug-target binding kinetics were not considered in the current model; this can be evaluated in the future to further strengthen and refine our modeling approach. Although it is challenging to obtain tissue samples from clinical studies, and it can be technically challenging to directly measure the lowering of free target with rapid turnover following treatment with mAbs (Zheng et al., 2015), any such data, even sparse, would allow significant improvement in the mPBPK model to better characterize the relationship between tissue site TE and clinical outcomes.

The current mPBPK model and TE-based analysis offers several advantages to inform forward- and reverse-translation in drug development as it 1) enables a mechanism- and physiology-based integration of multiple clinical datasets within a single framework, 2) can identify the extent of TE needed to exert comparable clinical responses to those seen with existing therapies, and 3) provide quantitative simulations to optimize drug-specific characteristics, including target binding affinity and clearance, for novel compounds in the discovery and preclinical stages sought to improve upon current therapies. Finally, the mechanistic nature of this model also enables utilization of physiologically relevant findings to other similar therapies.

## Conclusion

Clinical trial data from secukinumab and ixekizumab were analyzed quantitatively and simultaneously using MBMA and mPBPK modeling. The approach predicted the skin site TE achieved by the two drugs and the overall trend in the TE-clinical response relationship. By virtue of deriving several parameter values from (best available) sources in the literature, there is uncertainty associated with certain model components (e.g., tissue site IL-17A TE) in the absence of *in vivo* measurements. As such, it is important to restate that the main purpose of this work is to guide decision making for future drug development and the model predictions be updated as newer data emerge. While direct quantification of free IL-17A levels is challenging during treatment, the findings from this study reveal the value to assess tissue site TE and provide quantitative predictions to facilitate future drug development via IL-17A suppression in psoriasis.

## Data Availability

The original contributions presented in the study are included in the article/Supplementary Material, further inquiries can be directed to the corresponding author.
