# Supplementary material for: Minimal Physiologically-Based Pharmacokinetic (mPBPK) Metamodeling of Target Engagement in Skin Informs Anti-IL17A Drug Development in Psoriasis
Source: Front Pharmacol. 2022 Apr 25;13:862291. doi: 10.3389/fphar.2022.862291 (PMC9083543; doi:10.3389/fphar.2022.862291)
Supplement: Supplementary file 1 [file DataSheet2.DOCX]

;; 1. Based on: 2nd generation mPBPK-TE model for IL-17 (by VAyyar; based on Cao & Jusko, JPKPD, 2013)

;; 2. Description: secukinumab mPBPK-TE-MBMA

;; x1. Author: JLee

$PROBLEM secukinumab mPBPK

$INPUT ID STU TIME DV DVID AMT DOSE EVID MDV CMT ROUT

$DATA IL17_mPBPK_NM_v01_LN.csv IGNORE=#

;IGNORE=(DVID.EQ.3)

;IGNORE=(DVID.EQ.2)

$SUBROUTINES ADVAN6 TOL=5

;DVID= 0(dose), 1(serum PK), 2(skin PK), 3(total IL-17A in serum)

;CMT = 1(ABS), 2(serum), 3(muscle), 4(leaky), 5(skin), 6(lymph), 7(total IL17A serum), 8(total IL17A skin)

$MODEL

COMP = (SUBC) ;1

COMP = (CENT) ;2

COMP = (MUSC) ;3

COMP = (LEAK) ;4

COMP = (SKIN) ;5

COMP = (LYMP) ;6

COMP = (FISE) ;7

COMP = (FISK) ;8

;COMP = (TISE) ;9

;COMP = (TISK) ;10

$PK

TVCL = THETA(1)

CL = TVCL *EXP(ETA(1))

TVFA = THETA(2)

FA = TVFA *EXP(ETA(2))

TVKA = THETA(3)

KA = TVKA *EXP(ETA(3))

L = THETA(4)

LS = THETA(5)

L1 = THETA(6)

L2 = THETA(7)

TVVP = THETA(8)

VP = TVVP *EXP(ETA(4))

V2 = THETA(9)

VS = THETA(10)

V1 = THETA(11)

VL = THETA(12)

kdegSK = THETA(13)

kdegSE = THETA(14)

TVkintSK = THETA(15)

kintSK = TVkintSK *EXP(ETA(5))

TVkintSE = THETA(16)

kintSE = TVkintSE *EXP(ETA(6))

BSsk = THETA(17)

BSse = THETA(18)

KD = THETA(19)

TVsSK = THETA(20)

sSK = TVsSK *EXP(ETA(7))

s1 = THETA(21)

TVs2 = THETA(22)

s2 = TVs2 *EXP(ETA(8))

sL = THETA(23)

ksynSK = BSsk*kdegSK

ksynSE = BSse*kdegSE

A_0(7) = BSse

A_0(8) = BSsk

;CMT = 1(ABS), 2(serum), 3(muscle), 4(leaky), 5(skin), 6(lymph), 7(total IL17A serum), 8(total IL17A skin), 9(Drug-IL17A serum), 10(Drug-IL17A skin)

$DES

Csk_f = (0.5*((A(5) - A(8) - KD) + ((A(5) - A(8) - KD)**2 + 4*KD*A(5))**0.5))

Cse_f = (0.5*((A(2) - A(7) - KD) + ((A(2) - A(7) - KD)**2 + 4*KD*A(2))**0.5))

AR_sk = A(8)*Csk_f/(KD + Csk_f)

AR_se = A(7)*Cse_f/(KD + Cse_f)

DADT(1) = - KA*A(1)

DADT(2) = (KA*FA*A(1) + L*A(6) - L1*Cse_f*(1-s1) - L2*Cse_f*(1-s2) - LS*Cse_f*(1-sSK) - CL*Cse_f - (kintSE*AR_se*VP))/VP

DADT(3) = (Cse_f*L1*(1-s1) - A(3)*L1*(1-sL))/V1

DADT(4) = (Cse_f*L2*(1-s2) - A(4)*L2*(1-sL))/V2

DADT(5) = (Cse_f*LS*(1-sSK) - Csk_f*LS*(1-sL) - (kintSK*AR_sk*VS))/VS

DADT(6) = (Csk_f*LS*(1-sL) + A(3)*L1*(1-sL) + A(4)*L2*(1-sL) - L*A(6))/VL

DADT(7) = ksynSE - kdegSE*(A(7) - AR_se) - kintSE*AR_se

DADT(8) = ksynSK - kdegSK*(A(8) - AR_sk) - kintSK*AR_sk

$ERROR

IF(CMT.EQ.2) THEN

;IPRED = A(2)

;W = SQRT(THETA(24)**2*IPRED**2+THETA(25)**2) ; combined error model

IPRED = log(A(2)) ; using logC

W = SQRT(THETA(24)**2 + THETA(25)**2/IPRED**2) ;when using logC

IRES = IPRED - DV

IWRES = IRES/W

Y = IPRED + W*EPS(1)

ENDIF

IF(CMT.EQ.5) THEN

;IPRED = A(5)

;W = SQRT(THETA(26)**2*IPRED**2+THETA(27)**2) ; combined error model

IPRED = log(A(5)) ; using logC

W = SQRT(THETA(26)**2 + THETA(27)**2/IPRED**2) ;when using logC

IRES = IPRED - DV

IWRES = IRES/W

Y = IPRED + W*EPS(2)

ENDIF

IF(CMT.EQ.7) THEN

;IPRED = A(7)

;W = SQRT(THETA(28)**2*IPRED**2+THETA(29)**2) ; combined error model

IPRED = log(A(7)) ; using logC

W = SQRT(THETA(28)**2 + THETA(29)**2/IPRED**2) ;when using logC

IRES = IPRED - DV

IWRES = IRES/W

Y = IPRED + W*EPS(3)

ENDIF

$THETA

(0, 0.154) ; 1. CL, L/day

0.729 FIX ; 2. FA

0.18 FIX ; 3. KA, 1/day

2.9 FIX ; 4. L, L/day

0.247 FIX ; 5. LS, L/day

0.71 FIX ; 6. L1, L/day

1.943 FIX ; 7. L2, L/day

2.6 FIX ; 8. VP, L

4.368 FIX ; 9. V2, L

1.81 FIX ; 10. VS, L

6.3 FIX ; 11. V1, L

2.6 FIX ; 12. VL, L

2.44 FIX ; 13. kdegSK, 1/day

45.5 FIX ; 14. kdegSE, 1/day

0.34 FIX ; 15. kintSK, 1/day

(0, 1.2) ; 16. kintSE, 1/day

0.28 FIX ; 17. BSsk, pM

0.015 FIX ; 18. BSse, pM

129 FIX ; 19. KD, pM

(0, 0.63, 1) ; 20. sSK

0.95 FIX ; 21. s1

(0, 0.34, 1) ; 22. s2

0.2 FIX ; 23. sL

(0, 0.1) ; 24. PK,Serum PRO

0.000001 FIX ; 25. PK,Serum ADD

(0, 0.1) ; 26. PK,Skin PRO

0.000001 FIX ; 27. PK,Skin ADD

(0, 0.1) ; 28. TE PRO

0.000001 FIX ; 29. TE ADD

$OMEGA

(0, 0.1) ;1. CL

0 FIX ;2. FA

0 FIX ;3. KA

(0, 0.1) ;4. VP

0 FIX ;5. kintSK

0 FIX ;6. kintSE

0 FIX ;7. sSK

0 FIX ;8. s2

$SIGMA

1 FIX

1 FIX

1 FIX

$EST MAXEVAL=9999 PRINT=5 METHOD=1 INTER NOABORT NOTHETABOUNDTEST NOOMEGABOUNDTEST NOSIGMABOUNDTEST

$COV PRINT=E

;$SIM (1234) NSUBPROBLEMS=1 ONLYSIM

$TABLE ID TIME CMT DV IPRED PRED IWRES CWRES ONEHEADER NOPRINT FILE=sdtab1128

$TABLE ID CL kdegSK kdegSE kintSK kintSE sSK s2 ONEHEADER NOPRINT FILE=patab1128

;$TABLE ID SEX RACE IR IRPT PTITER STITER ONEHEADER NOPRINT FILE=catab1001

;$TABLE ID AGE WT HT CRT CRCL eGFR LBM BMI BSA ALB AST ALT ALP TB TP GGT LDH BSCD ONEHEADER NOPRINT FILE=cotab1001
