# Supplementary material for: Minimal Physiologically-Based Pharmacokinetic (mPBPK) Metamodeling of Target Engagement in Skin Informs Anti-IL17A Drug Development in Psoriasis
Source: Front Pharmacol. 2022 Apr 25;13:862291. doi: 10.3389/fphar.2022.862291 (PMC9083543; doi:10.3389/fphar.2022.862291)
Supplement: Supplementary file 2 [file DataSheet1.DOCX]

Minimal physiologically based-pharmacokinetic (mPBPK) meta-modeling of target engagement in skin informs anti-IL17A drug development in psoriasis

Vivaswath S. Ayyar, Jong Bong Lee, Weirong Wang, Meghan Pryor, Yanli Zhuang, Thomas Wilde, An Vermeulen

Frontiers in Pharmacology

**Minimal PBPK Model Differential Equations**

$$\frac{{dSC}_{depot}}{dt}=-k_{a}\cdot{SC}_{depot} {SC}_{depot}\left( 0 \right)=Dose (1)$$

$$\frac{{dC}_{p}}{dt}=\frac{{Input+C}_{lymph}\cdot L-C_{f}\cdot L_{1}\cdot\left( 1-\sigma_{1} \right){- C}_{f}\cdot L_{2}\cdot\left( 1-\sigma_{2} \right){- C}_{f}\cdot L_{s}\cdot\left( 1-\sigma_{s} \right){-C}_{f}\cdot CL-k_{int,p}\cdot{AR}_{p}\cdot V_{p}}{V_{p}}$$

$$Input=Dose \left( IV \right) or k_{a}\cdot{SC}_{depot}\cdot F C_{p}\left( 0 \right)=\frac{Dose}{V_{p}} \left( IV \right) or 0 (SC) (2)$$

$$\frac{{dC}_{mus}}{dt}=\frac{C_{f}\cdot L_{1}\cdot\left( 1-\sigma_{1} \right)-C_{mus}\cdot L_{1}\cdot\left( 1-\sigma_{L} \right)}{V_{mus}} C_{mus}\left( 0 \right)=0 (3)$$

$$\frac{{dC}_{leaky}}{dt}=\frac{C_{f}\cdot L_{2}\cdot\left( 1-\sigma_{2} \right)-C_{leaky}\cdot L_{2}\cdot\left( 1-\sigma_{L} \right)}{V_{leaky}} C_{leaky}\left( 0 \right)=0 (4)$$

$$\frac{{dC}_{sk}}{dt}=\frac{C_{f}\cdot L_{sk}\cdot\left( 1-\sigma_{s} \right)-C_{sk\_f}\cdot L_{s}\cdot\left( 1-\sigma_{L} \right)-k_{int,sk}\cdot{AR}_{sk}\cdot V_{sk}}{V_{sk}} C_{sk}\left( 0 \right)=0 (5)$$

$$\frac{{dC}_{lymph}}{dt}=\frac{C_{sk\_f}\cdot L_{sk}\cdot\left( 1-\sigma_{sk} \right)+C_{mus}\cdot L_{mus}\cdot\left( 1-\sigma_{1} \right){+C_{leaky}\cdot L_{2}\cdot\left( 1-\sigma_{2} \right)-C}_{lymph}\cdot L}{V_{lymph}} C_{lymph}\left( 0 \right)=0 (6)$$

where *SC_depot_* is the amount of therapeutic monoclonal antibody (mAb) at the SC injection site and *k_a_* is the first-order absorption rate constant for mAb. C_p_ and C_f_ are the total and free concentrations of mAb in plasma, C_sk_ and C_sk_f_ are the total and free concentrations of mAb in skin, and C_mus_ and C_leaky_ are concentrations of mAb in interstitial fluid (ISF) in skeletal muscle and a lumped tissue mass comprising fenestrated vascular endothelium, and C_lymph_ is the concentration of mAb in lymph. The V_mus_ and V_skin_ are the calculated ISF volumes of muscle and skin and V_leaky_ (0.35·ISF·K_p_, where K_p_ is the available fraction of ISF for antibody distribution) is the ISF volume of the lumped ‘leaky’ tissues. The V_lymph_ is lymph volume, which is assumed equal to blood volume. The L is total lymph flow rate; where L_1_ and L_s_ account for 1/3 of the total lymph flow and L_2_ accounts for 2/3 of the total lymph flow. The σ_s,_ σ_1,_ and σ_2_ are vascular reflection coefficients for skin, muscle, and leaky tissues. The σ_L_ is the lymphatic capillary reflection coefficients and is assumed to be 0.2. CL is the linear clearance of mAb.

$$\frac{{dR}_{tot,p}}{dt}=k_{syn}-k_{deg,p}\cdot{(R}_{tot,p}-{AR}_{p})-k_{int,p}\cdot{AR}_{p} R_{tot,p}\left( 0 \right)=\frac{k_{syn}}{k_{deg,p}} (7)$$

$$\frac{{dR}_{tot,sk}}{dt}=k_{syn}-k_{deg,sk}\cdot{(R}_{tot,sk}-{AR}_{sk})-k_{int,sk}\cdot{AR}_{sk} R_{tot,sk}\left( 0 \right)=\frac{k_{syn}}{k_{deg,sk}} (8)$$

The drug-target interactions of the mAbs and IL-17A in serum and skin were characterized using TMDD kinetics. *R_tot,p_* and *R_tot,sk_*  are the total IL-17 and A*R_p_* and A*R_sk_*  are the mAb-IL-17A complex concentrations in serum and skin, respectively. The *k_syn_* is a zero-order rate constant for IL-17 biosynthesis, whereas *k_deg,p_* and *k_deg,sk_*  are the first-order rate constants for IL-17 elimination in serum and skin. *k_int,p_* and *k_int,sk_*  refers to the elimination rate constants of mAb-IL-17A complex in serum and skin.

Assuming quasi-equilibrium conditions, *C_f_* and C_sk_f_ can be described as

$C_{f}=0.5\cdot\left[ \left( C_{p}-K_{D}-R_{tot,p} \right)+\sqrt{\left( C_{p}-K_{D}-R_{tot,p} \right)^{2}+4\cdot C_{p}\cdot K_{D}} \right]$ (9)

$C_{sk\_f}=0.5\cdot\left[ \left( C_{sk}-K_{D}-R_{tot,sk} \right)+\sqrt{\left( C_{sk}-K_{D}-R_{tot,sk} \right)^{2}+4\cdot C_{sk}\cdot K_{D}} \right]$ (10)

Where *K_D_* refers to the equilibrium dissociation constant of each drug with IL-17A. A*R_p_* and A*R_sk_*  can be described as:

$${AR}_{P}=\frac{R_{tot,p}+C_{f}}{K_{D}+C_{f}} (11)$$

$${AR}_{sk}=\frac{R_{tot,sk}+C_{sk\_f}}{K_{D}+C_{sk\_f}} (12)$$
